# Acute Urticaria as the First Symptom of COVID-19: A Case Report

**DOI:** 10.7759/cureus.20806

**Published:** 2021-12-29

**Authors:** Diego M Watashi, Diogo R Sene, Júlia B Garófalo, Renan H Merlini, Alexandre B Merlini

**Affiliations:** 1 Internal Medicine, Universidade de Mogi das Cruzes, Mogi das Cruzes, BRA; 2 Intensive Care Unit, Hospital Dr. Arnaldo Pezzuti Cavalcanti, Mogi das Cruzes, BRA; 3 Medicine, Universidade Nove de Julho, Osasco, BRA; 4 Emergency Department, Grupo NotreDame Intermédica, São Paulo, BRA

**Keywords:** cutaneous manifestations, skin lesions, urticaria, sars-cov-2, covid-19, case report

## Abstract

During the SARS-CoV-2 pandemic, various rashes associated with COVID-19 infection have been reported, including urticaria. Urticaria is a limited and usually benign condition, presenting as pruritic wheals, with or without edema. A 39-year-old woman presented with a pruritic rash on her arms spreading to her trunk and face over two days, followed by headache, nausea, vomiting, abdominal discomfort, diarrhea, myalgia, arthralgia, anosmia, and dyspepsia for three days. Fever, dry cough, and odynophagia started on the day of the consult. The patient had a history of hypertension but denied a history of atopic conditions, similar previous presentations, or recent ingestion of new medications. SARS-CoV-2 COVID-19 PCR testing was positive. She was prescribed oral antihistamine for the itching and was discharged. During a follow-up after two weeks, the patient was asymptomatic with complete resolution of the rash on day 7 of symptoms. Knowing the cutaneous manifestations of COVID-19 can aid in the early identification of this disease and prevent misdiagnosis. The presence of cutaneous manifestations in COVID-19 is suggested to be related to disease severity, but data are needed to study any prognostic value of dermatologic manifestations in COVID-19.

## Introduction

During the SARS-CoV-2 pandemic, a variety of rashes associated with COVID-19 infection have been reported. The most common cutaneous manifestations include maculopapular exanthema, urticarial lesions, and vesicular eruptions [1].

Urticaria is a limited and usually benign condition, caused by immunoglobulin E or non-immunoglobulin E mediated mast cell and basophil activation and histamine release. It presents as pruritic wheals, with or without edema [2]. In COVID-19, the lesions are mainly located on the face and upper body and are rarely seen before the typical symptoms of the disease (fever, cough, fatigue) [1].

In our case, we describe a patient without new medication use presenting with acute prodromal urticaria before typical COVID-19 manifestations.

## Case presentation

**Figure 1:**
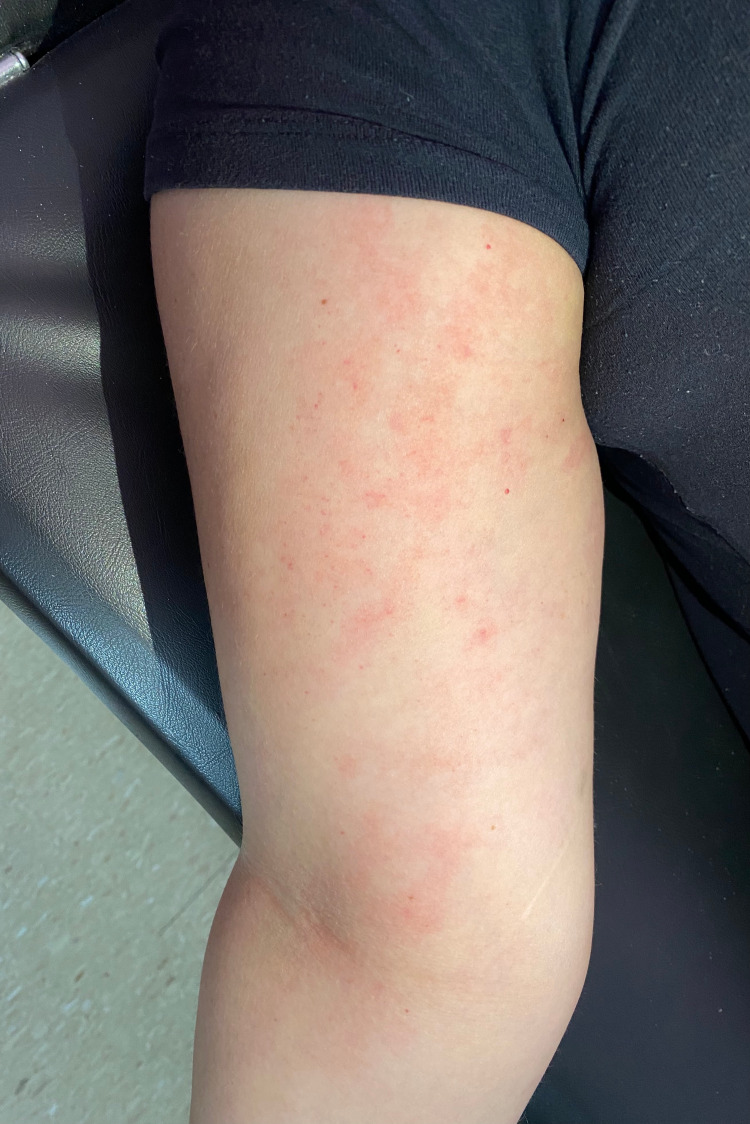
Initial urticarial rash on the left upper limb.

**Figure 2:**
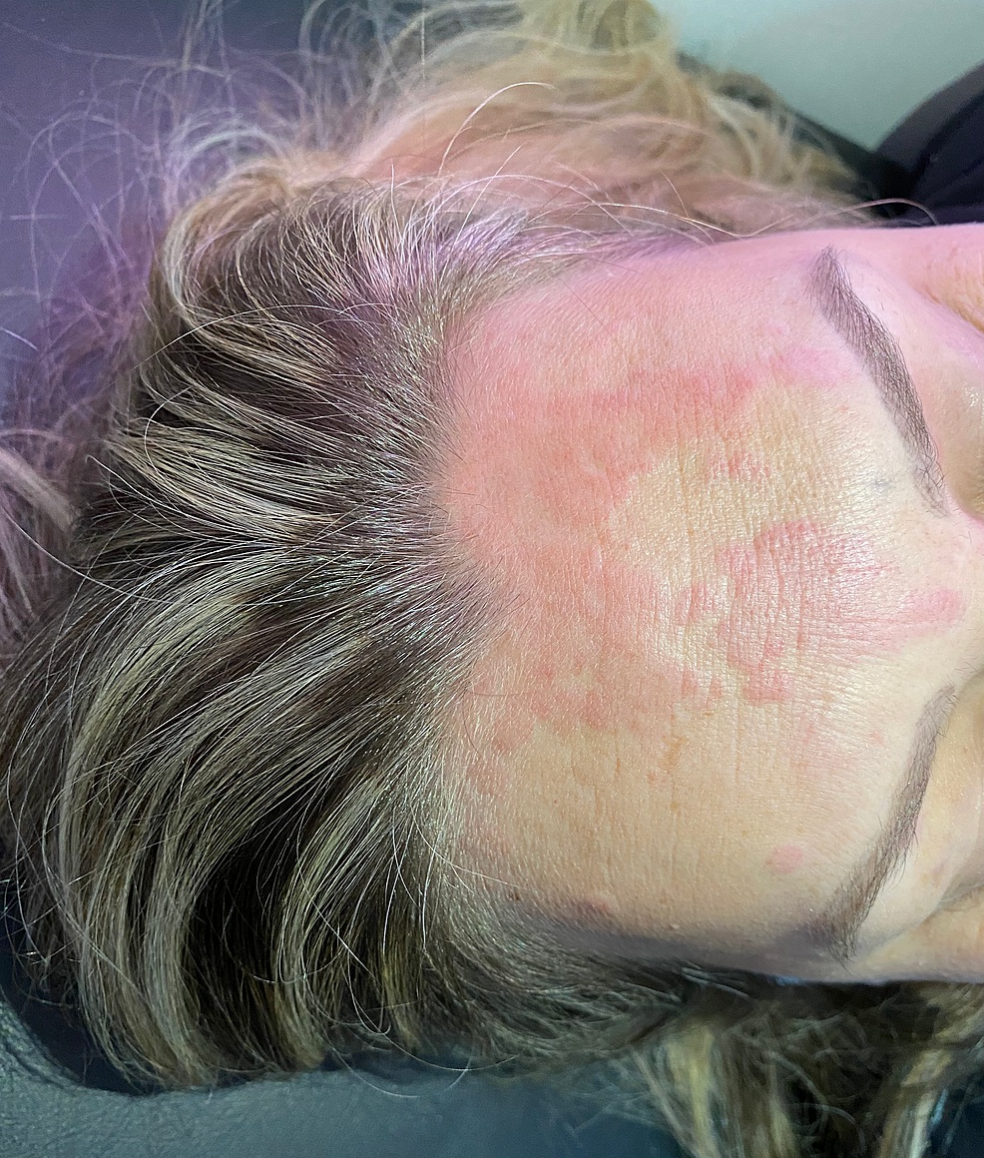
Erythematous plaques on the forehead.

**Figure 3:**
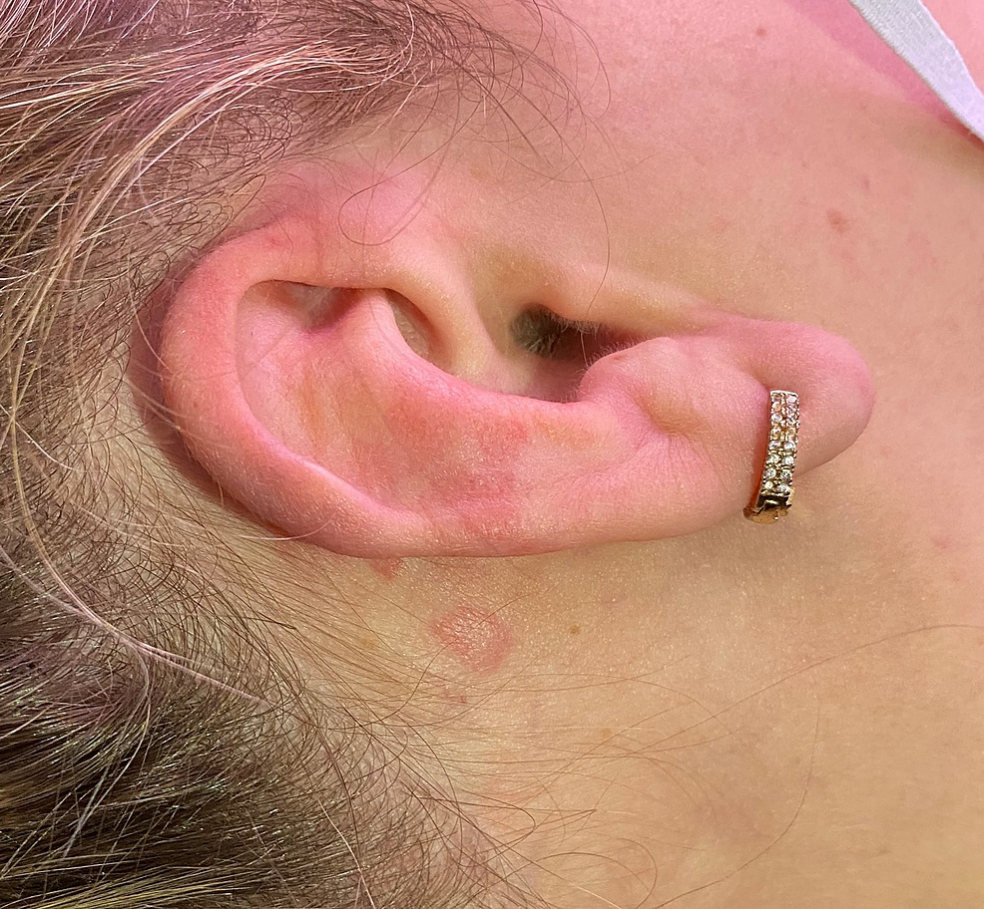
Erythematosus plaques on the right ear.

**Figure 4:**
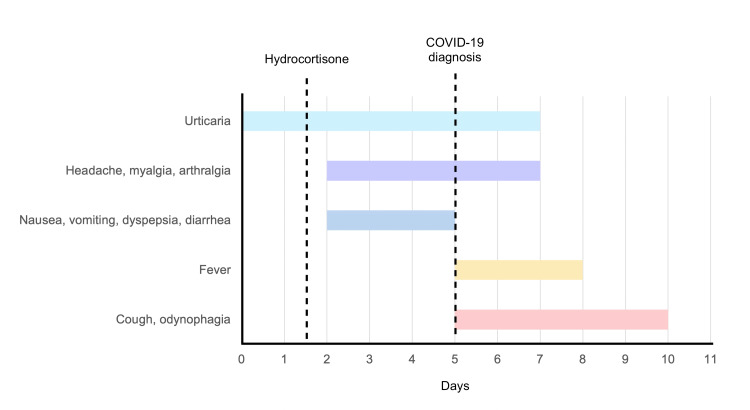
Timeline of symptoms and events.

A 39-year-old woman presented to the emergency room complaining of a pruritic rash over her face, arms, and trunk. The rash first started on her arms (Figure 1) five days ago and spread to her trunk and face (Figures 2, 3) over the next days. The patient sought medical care four days ago and was medicated with intravenous hydrocortisone, improving the itching but without improvement of the rash. Three days ago, she developed headache, nausea, vomiting, abdominal discomfort, diarrhea, myalgia, arthralgia, anosmia, and dyspepsia. She denied dyspnea. A few hours ago, the patient started to complain of fever (39°C), dry cough, and odynophagia (Figure 4).

The patient had a history of hypertension treated with losartan and is allergic to promethazine and dexchlorpheniramine; she denied a history of atopic conditions, similar previous presentations, recent ingestion of new medications, or contact with any individuals with a confirmed positive case of COVID-19. Her family history was non-contributory.

On examination, erythematous plaques were visible over her arms and chin. Oral cavity examination showed erythematous tonsils. Respiratory examination showed mild crackles over the lungs bases, with no signs of respiratory distress. The patient was noted to be obese (BMI: 34.6). Her vital signs were within normal limits. Computed tomography (CT) was performed and small ground-glass opacities were observed scattered across the two lungs, affecting probably less than 25% of lung parenchyma. SARS-CoV-2 COVID-19 PCR testing was positive. She was prescribed oral antihistamine for the itching and discharged home with instructions regarding quarantine.

During a follow-up after two weeks, the patient was asymptomatic with complete resolution of the rash on day 7 of the symptoms.

## Discussion

Since the first COVID-19 report, clinical manifestations were described in most cases as fever, nonproductive cough, dyspnea, myalgia, fatigue, and normal or decreased leukocyte count, in addition to radiographic evidence of ground-glass opacities on the chest CT scan. However, with the growing COVID-19 data around the world, different presentations have been reported, including cutaneous manifestations of this disease [1].

The presence of cutaneous manifestations in COVID-19 has been reported to range from 0.2% to 29% of cases [1]. The presentations include vesicles, chilblain lesions, exanthems, maculopapules, urticaria, petechiae, erythema multiforme-like lesions, livedo, and necrosis [1,3].

The pathophysiology of urticaria in COVID-19 is still unclear. It was thought that the skin manifestation was drug-mediated by COVID-19 therapy [4]. However, many case reports, including this one, describe skin changes before medication use. The urticaria may be mediated by the acute systemic inflammatory response to SARS-CoV-2 acute infection, causing pro-inflammatory cytokines release and mast cell activation [4].

Dermatologic manifestations rarely appear before primary COVID-19 symptoms, happening in only 5.9% of COVID-19 cases with skin lesions [1]. In those particular situations, the prompt identification of COVID-19 skin lesions can aid in the early diagnosis of the disease.

The presence of cutaneous manifestations in COVID-19 is suggested to be related to disease severity. A Spanish study correlated the feature of urticarial or maculopapular lesions to more severe disease [3]. A Chinese study found that urticaria was more common in severe COVID-19 patients, been observed in 1.2% of nonsevere patients and 1.7% of severe patients, yet without statistical significance [5]. However, Sachdeva et al. stated an unlikely correlation between skin lesions and disease severity in their literature review with 21 patients [6]. Further studies (ideally, cohort studies) are needed in the future to determine the prognostic value of prodromal dermatologic manifestations in COVID-19.

Skin manifestations are usually self-limited, as urticaria lasts around 6.8 days and up to 10 days [3,6]. The treatment for urticaria is focused on symptoms improvement with antihistamines and low-dose corticosteroids in individual cases (lack of improvement with antihistamines only). Most patients achieve adequate symptom control with antihistamines only and may continue the medication until the urticaria resolution [4]. There are no specific guidelines for urticaria or other cutaneous manifestations associated with COVID-19.

The main limitation to be considered in this case report is the small sample size, as no strong conclusion can be drawn and applied to patient care and prognosis. However, this case report serves as literature evidence for further studies.

## Conclusions

During the SARS-CoV-2 pandemic, health care workers must maintain a high clinical suspicion of COVID-19 when examining patients with atypical presentations. Knowing the cutaneous manifestations of COVID-19 can aid in the early diagnosis and treatment of the disease and prevent infectious spread and serious outcomes. More studies are needed to determine any prognostic value of dermatologic manifestations in COVID-19.
